# Deregulated Sex Chromosome Gene Expression with Male Germ Cell-Specific Loss of *Dicer1*

**DOI:** 10.1371/journal.pone.0046359

**Published:** 2012-10-04

**Authors:** Anne R. Greenlee, Meng-Shin Shiao, Elizabeth Snyder, F. William Buaas, Tongjun Gu, Timothy M. Stearns, Manju Sharma, Elizabeth P. Murchison, Gabriella C. Puente, Robert E. Braun

**Affiliations:** 1 The Jackson Laboratory, Bar Harbor, Maine, United States of America; 2 Wellcome Trust Sanger Institute, Hinxton, United Kingdom; 3 Yale University, New Haven, Connecticut, United States of America; University of Muenster, Germany

## Abstract

MicroRNAs (miRNAs) are a class of endogenous, non-coding RNAs that mediate post-transcriptional gene silencing by inhibiting mRNA translation and promoting mRNA decay. DICER1, an RNase III endonuclease encoded by *Dicer1*, is required for processing short 21–22 nucleotide miRNAs from longer double-stranded RNA precursors. Here, we investigate the loss of *Dicer1* in mouse postnatal male germ cells to determine how disruptions in the miRNA biogenesis pathway may contribute to infertility. Reduced levels of *Dicer1* transcripts and DICER1 were confirmed in germ cell knock-out (GCKO*)* testes by postnatal day 18 (P18). Compared to wild-type (WT) at 8 weeks, GCKO males had no change in body weight; yet showed significant reductions in testis mass and sperm number. Histology and fertility tests confirmed spermatogenic failure in GCKO males. Array analyses at P18 showed that in comparison to WT testes, 75% of miRNA genes and 37% of protein coding genes were differentially expressed in GCKO testes. Among these, 96% of miRNA genes were significantly down-regulated, while 4% miRNA genes were overexpressed. Interestingly, we observed preferential overexpression of genes encoded on the sex chromosomes in GCKO testes, including more than 80% of previously identified targets of meiotic sex chromosome inactivation (MSCI). Compared to WT, GCKO mice showed higher percentages of germ cells at early meiotic stages (leptotene and zygotene) but lower percentages at later stages (pachytene, diplotene and metaphase I) providing evidence that deletion of *Dicer1* leads to disruptions in meiotic progression. Therefore, deleting *Dicer1* in early postnatal germ cells resulted in deregulation of transcripts encoded by genes on the sex chromosomes, impaired meiotic progression and led to spermatogenic failure and infertility.

## Introduction

MicroRNAs (miRNAs) are single-stranded, non-coding (nc) RNAs with an average length of 21–23 base pairs (bp). They have been identified on all mammalian chromosomes except the Y and usually function as posttranscriptional regulators through base-pairing with the untranslated regions of targeted messenger RNAs (mRNAs) [1], [2]. More than 800 miRNA genes have been discovered by sequencing small RNA libraries from a variety of organisms and tissues, including 55 miRNAs detected highly, exclusively or predominantly in the adult mouse testis [3], [4]. MiRNAs play critical roles in biological processes such as cell proliferation, differentiation and apoptosis [5]. DICER1, an RNase III endonuclease, is an important component of miRNA biogenesis as it cleaves the hairpin structure of double-stranded precursor miRNA (pre-miRNA) into a ∼23 nucleotide miRNA:miRNA* duplex. The duplex is unraveled and the single-stranded, mature miRNA is loaded into the RNA induced silencing complex (RISC). Binding of miRNAs in RISC to a specific mRNA transcript, negatively regulates gene expression by either reducing translational efficiency or by promoting mRNA degradation [6], [7], [8].

Spermatogenesis is a complex, multistep process that leads diploid undifferentiated spermatogonia through mitotic proliferation, meiosis (spermatocytes) and differentiation of haploid gametes (spermatids) [9]. Growing evidence suggests that miRNAs play fundamental roles during spermatogenesis. Robust expression of miRNAs has been detected in the normal testes at postnatal day 7 (P7) and 14 (P14) encoded by genes on chromosome 12 and the X chromosome, respectively [10]. Several of these miRNAs show developmentally regulated expression suggesting a role of these molecules in male germ cell development. When a germ cell-specific miRNA, miR-34c, was over-expressed in HeLa cells, the non-germ cell transcriptome was shifted toward a germinal lineage expression profile [11]. In germ cell nuclei following meiosis, histones are replaced by transition protein 1 and 2 (TNP1 and TNP2), which are in turn replaced by protamine 1 and 2 (PRM1 and PRM2) in elongating spermatids [12]. Yu et al. [13] showed that miR-122a binds to recognition sites on transition protein 2 (*Tnp2*) mRNA in late-stage germ cells and modulates temporal translation and degradation of stored transcripts. This finding of miR-122a binding recognition sites on *Tnp2* mRNA uncovers a possible role for miRNAs in post meiotic temporal transcriptional control.

Mouse models with conditional deletion of *Dicer1* in the testis have demonstrated the importance of DICER1 and *Dicer1*-dependent small RNAs in the regulation of male germ cell development. Primordial germ cell-specific deletion of *Dicer1* by cre recombinase driven by the promoter of tissue-nonspecific alkaline phosphatase (TNAP-cre) from embryonic day 10 (E10) onwards showed that miRNAs are necessary in the proliferation and early differentiation of the male gonocytes [14]. However, low efficiency and ectopic expression of TNAP-cre and recombination during embryogenesis make it difficult to interpret spermatogenesis defects in the adult. Deletion of *Dicer1* in type A spermatogonia by *Neurog3* promoter-driven cre (*Ngn3*-cre) resulted in defective spermatogenesis and infertility characterized by the arrest of spermatid elongation prior to the histone-protamine exchange [15]. Conditional deletion of *Dicer1* using *Ddx4-*cre in spermatogonia at E18 produced abnormalities in seminiferous tubules appearing as early as P15 with increased germ cell vacuolization, apoptosis and disorganization [16]. Moreover, deletion of *Dicer1* with retinoic acid gene 8-driven cre (*Stra8*-cre) impaired spermiogenesis at the round spermatid stage [17]. However, these reports leave open questions about the large-scale effects of *Dicer1* inactivation on miRNA biogenesis and the impacts on the postnatal testis transcriptome, meiotic progression and translational control.

To more thoroughly characterize the function of miRNAs during spermatogenesis, we conditionally deleted *Dicer1* in postnatal spermatogonia and examined the impact on male germ cell development as well as the expression profiles of miRNA genes and protein coding genes. Early postnatal germ cell-specific loss of *Dicer1* resulted in spermatogenic failure and infertility associated with down-regulation of testis miRNAs, deregulation of the testis transcriptome and preferential overexpression of sex chromosome genes. Thus, miRNAs are critical for normal testicular development.

## Materials and Methods

### Mice

The Jackson Laboratory Animal Care and Use Committee approved all animal studies (Permit Number: #07007). Studies were carried out in strict accordance with the recommendations in the National Academy of Science *Guide for the Care and Use of Laboratory Animals* (1996; revised 2011).

**Table 1:** Primers used for q-RT-PCR validation of Affymetrix Mouse Gene 1.0 ST arrays and *Dcr* RNase III transcript levels.

ActB 70F	CCA GTT CGC CAT GGA TGA CGA TAT			
ActB 277R	GTC AGG ATA CCT CTC TTG CTC TG			
Sox9 862F	TGC AGC ACA AGA AAG ACC AC			
Sox9 1141R	CCC TCT CGC TTC AGA TCA AC			
Stra8 271F	AGT CTG ATA TCA CAG CCT CAA AG			
Stra8 450R	CAT TCT CGG AAT ACA TTC TGG CA			
Adad1 1F	TAC AGG GAG CCT TGC TGA GT			
Adad1 1R	TGA TGT GAG TGC GTC ATC AA			
Bcl2l11 4F	CCA GCC CTG GCC CTT TTG CTA			
Bcl2l11 4R	TCC GGG CGC AGA TCT TCA GG			
Kitl 2F	CCA TGG CAT TGC CGG CTC TCA			
Kitl 2R	ACCAGC CAC TGT GCG AAG GTA A			
Vsig4 3F	GGC CGC CTG AAA GTG AGC CA			
Vsig4 3R	AGG AGT GCA GGG TTG TAG GTG CT			
Pgrmc1 1F	AGT TCT ACG GGC CTG AGG GGC			
Pgrmc1 1R	AGG CTC CTC CCC TTC CTT CAG C			
Lamp2 1F	TGG CTA ATG GCT CAG CTT TCA ACA			
Lamp2 1R	CCC ACC GCT ATG GGC ACA AGG			
Ube1y1 218F	GCC ATA GTT TTC TGC TCG GA			
Ube1y1 115R	TTG GAT TCA AGA TGT ACC CCA			
Dicer 21F	TAA CCT GGA GCG GCT TGA GA			
Dicer 24R	CAG GAA TTC TAA GCG CTG GT			

The genetic background of mice used in this study was mixed FVB and 129S1. Mutant and wild type (WT) mice were bred in the research colony of Dr. R.E. Braun. A gene cassette with the promoter of germ cell-specific *Stra8* that drives the activity of cre recombinase [*Stra8*-icre; formally Tg(Stra8-cre^1Reb^)] was used to excise *Dicer1* in the germ cells [18]. A modified *Dicer1* ^Floxed^ allele (*Dicer1* ^tm1Smr^) was generated by inserting two lox-P sites flanking exons 22 and 23 of *Dicer1* [19]. To generate conditional *Dicer1^−/−^* male mice, we first mated heterozygous *Dicer1* ^Floxed/WT^ males carrying the *Stra8*-icre transgene with homozygous females carrying two *Dicer1* floxed alleles (*Dicer* ^Floxed/Floxed^). Male and female offspring carrying a *Dicer1* conditional allele and the *Stra8*-icre transgene (*Dicer1* ^Floxed/WT^; *Stra8*-icre+) were intercrossed to generate male mice with the genotype (*Dicer1* ^Floxed/excised^ *; Stra8*-icre+). These males have *Dicer1^−/−^* germ cells and will henceforth be referred to as *Dicer1* germ cell knock out (GCKO) male mice. Excision of *Dicer1* from germ cells was confirmed by measuring *Dicer1* transcript and DICER1 protein levels in WT and GCKO testes.

**Figure 1:**
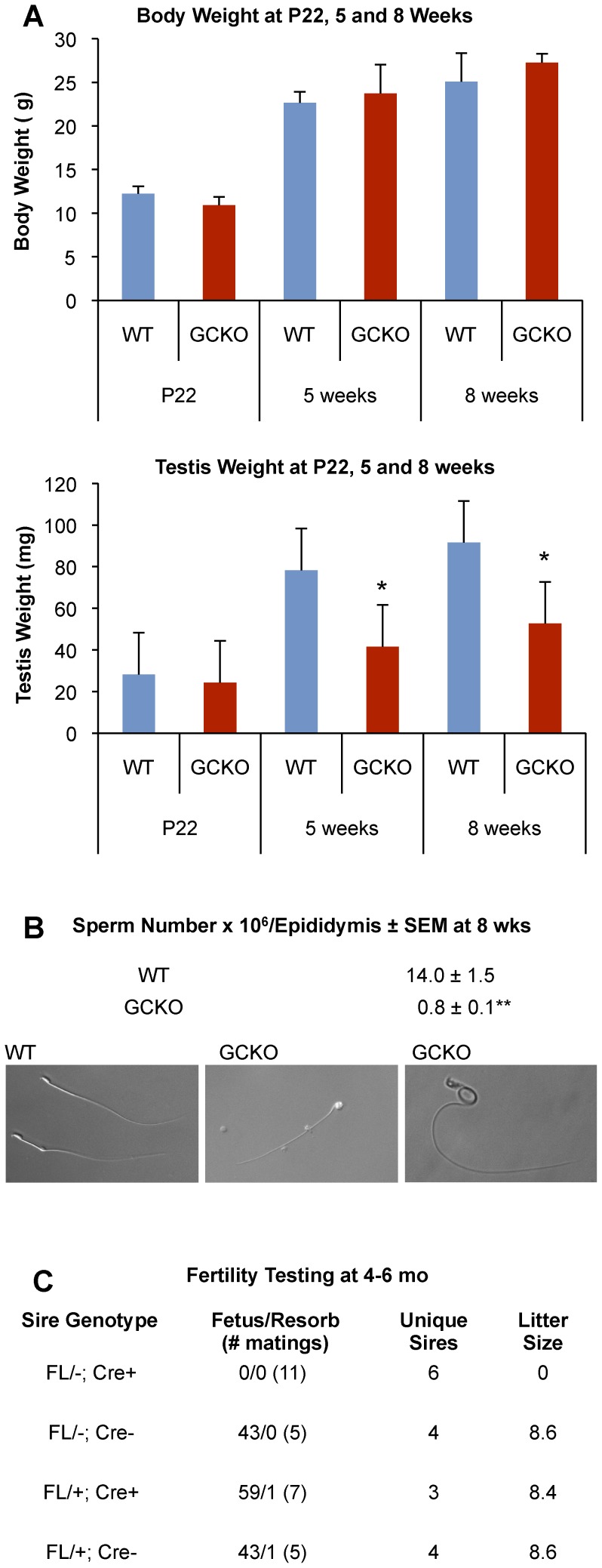
GCKO mice show reduced testis weight and sperm number, abnormal sperm morphology and infertility. (A) Body and testis weights were measured in WT and GCKO males at P22, 5 and 8 weeks (at each time point, n = 5 for WT and n = 5 for GCKO). In comparison to WT, GCKO males had no change in body weight over 8 weeks, yet showed significant reductions in testis weight as early as 5 weeks and remained so at 8 weeks (*both *P*≤0.004). Values represent the mean ± SEM. (B) In comparison to WT, GCKO epididymal sperm counts revealed significant reductions in sperm number by 8 weeks (**P  = 0.0003). Approximately 80% of WT and less than 10% of GCKO epididymal sperm showed normal head and tail morphologies as represented in the photomicrographs taken at 40x. (C) Fertility testing showed that 4–6 month old GCKO males were unable to sire litters in comparison to littermate controls when mated 3–6 times with 12-week fertile B6CBAF1/J females.

**Figure 2:**
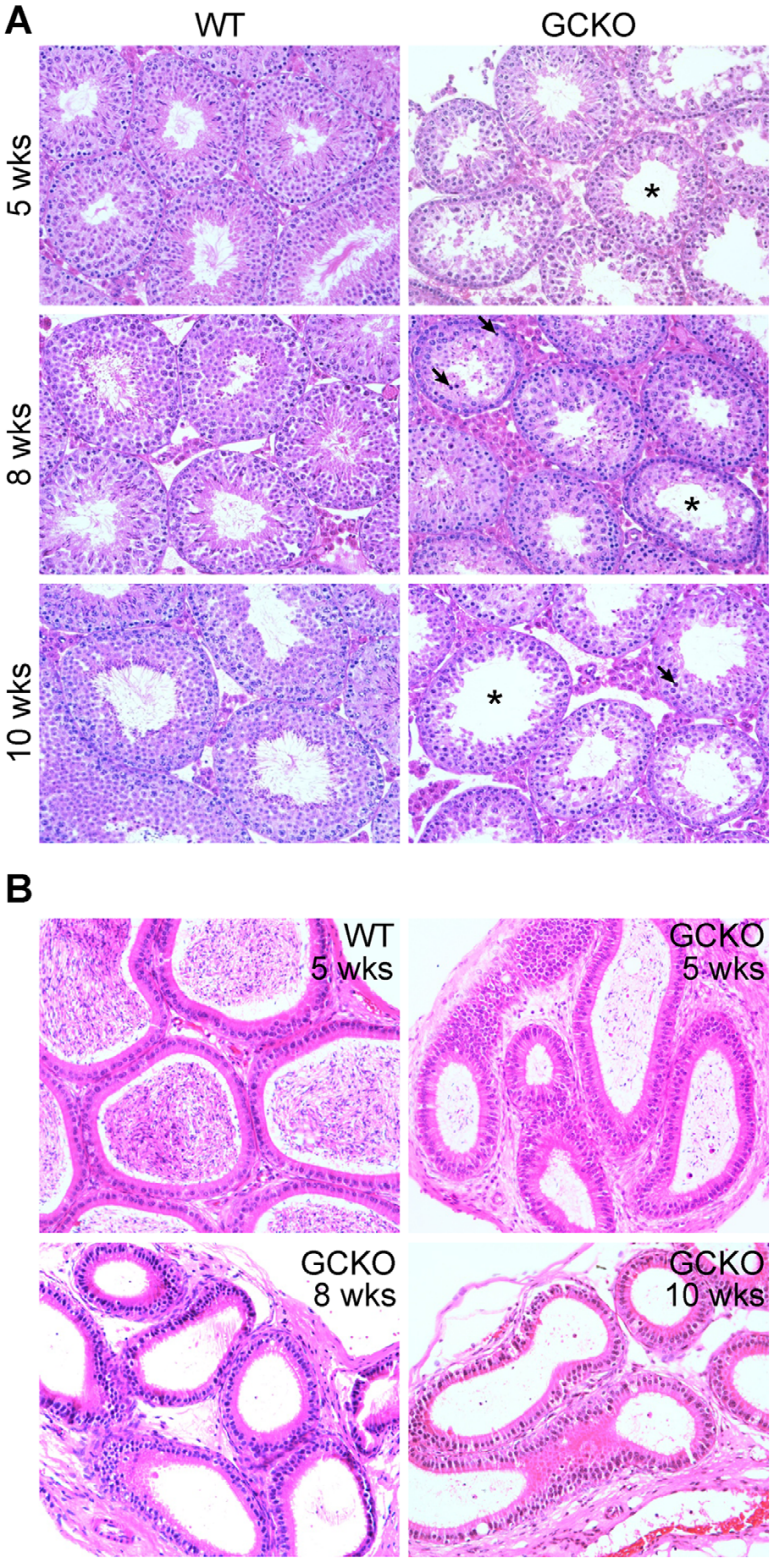
Histology comparing WT and GCKO seminiferous tubules and epididymides at 5, 8 and 10 weeks. (A) In comparison to WT seminiferous tubules at 5 weeks, GCKO sections show tubules with increased lumen diameters and few elongating and elongated spermatids (*). By 8 weeks, cross sections from GCKO testes show prominent pynotic cells (arrows) and reduced numbers of elongating and elongated spermatids. By 10 weeks, cross sections of GCKO testes show further enlargement of tubule lumens and absence of elongating spermatids in the majority of tubules. (B) Epididymides of WT mice at 5 weeks and GCKO mice at 5, 8 and 10 weeks showing reduced sperm numbers in epididymides of knockout mice by 5 weeks and the absence of mature spermatozoa in GCKO epididymides at the 8 and 10 week timepoints.

### Sperm Counts and Fertility Assessment

Epididymal sperm counts were evaluated at 8 weeks from sperm released into PBS from the cauda epididymis and ductus deferens during one hour of incubation at 37°C. Dilutions (1∶10) were prepared in 4% paraformaldehyde in PBS and sperm counts were performed using a hemocytometer. Body and testis weights were determined at P22, 5 and 8 weeks. Fertility testing was accomplished by 3–6 unique matings of WT and GCKO males with 10–12 week old females (B6D2F1) and determining the number of fetuses or resorptions at E12.5 for each mating pair. Phenotypic data (body and testis weights, sperm number) WT and GCKO samples are shown as a mean ± SEM. Data were analyzed by two-tailed Student *t*-test using JMP10 software with *P*≤0.05 considered significant.

**Figure 3:**
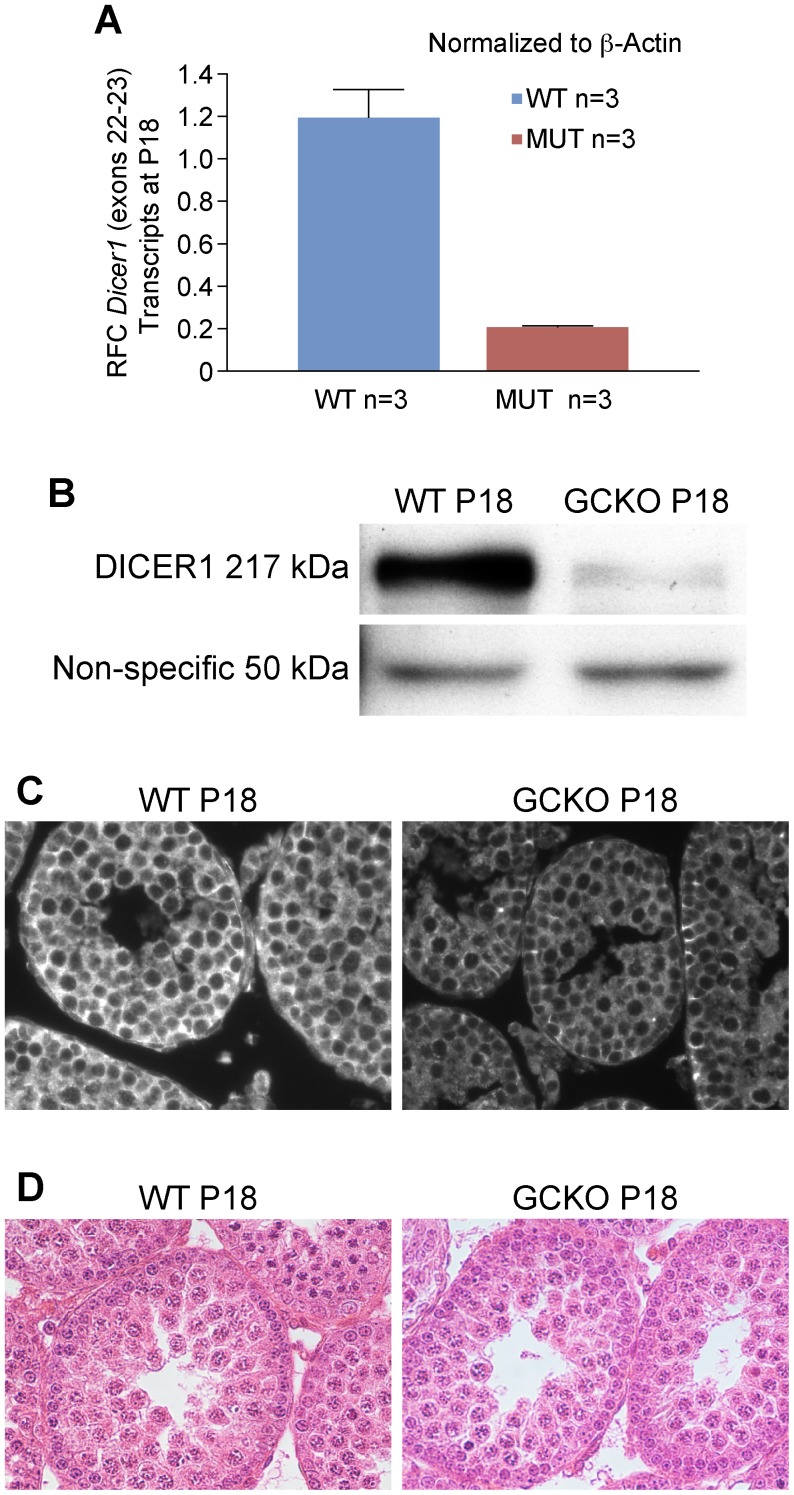
Levels of *Dicer1* transcripts and DICER1 protein in GCKO testis samples are significantly reduced by P18. (A) In comparison to WT testes, real-time qRT-PCR shows a 6-fold reduction in *Dicer1* RNase III endonuclease transcripts in GCKO testes by P18 (*P  = 0.018). (B) Western blot analysis using rabbit antibody against the N-terminal helicase domain of DICER1 and HRP-conjugated goat anti-rabbit IgG antibody shows reduced protein expression in GCKO testes by P18. The arrows point to the 217 kDa DICER1 protein and to the equivalent loading of 50 kDa protein from WT and GCKO testes. (C) Immunofluorescence detection of DICER1 protein in WT and GCKO testes at P18 using the same antibody used for western blotting. In WT sections, DICER1 localizes to the cytoplasm of most cell types populating the P18 testis, including Sertoli cells, spermatogonia and spermatocytes. At this developmental time point, secondary spermatocytes and spermatids are not present. In the P18 GCKO testis sections, DICER1 appears primarily in the cytoplasm of Sertoli cells located near the basement membrane with scant detection of DICER1 protein in the cytoplasm of spermatogonia and pachytene spermatocytes (magnification  = 40x). (D) Cross sections of WT and GCKO testes on P18 stained with HE show no major differences in cellular composition suggesting that the changes in in *Dicer1* transcript and protein levels in the GCKO testes were not explained by differences in cell populations.

### Histology

Testes retrieved from GCKO mice and wild-type control litter mates at P18 and at 5, 8 and 10 weeks were fixed in Bouin’s, embedded in paraffin and sectioned. Sections were deparaffinized in xylene, rehydrated and stained with hematoxylin and eosin for phenotype determination.

**Figure 4:**
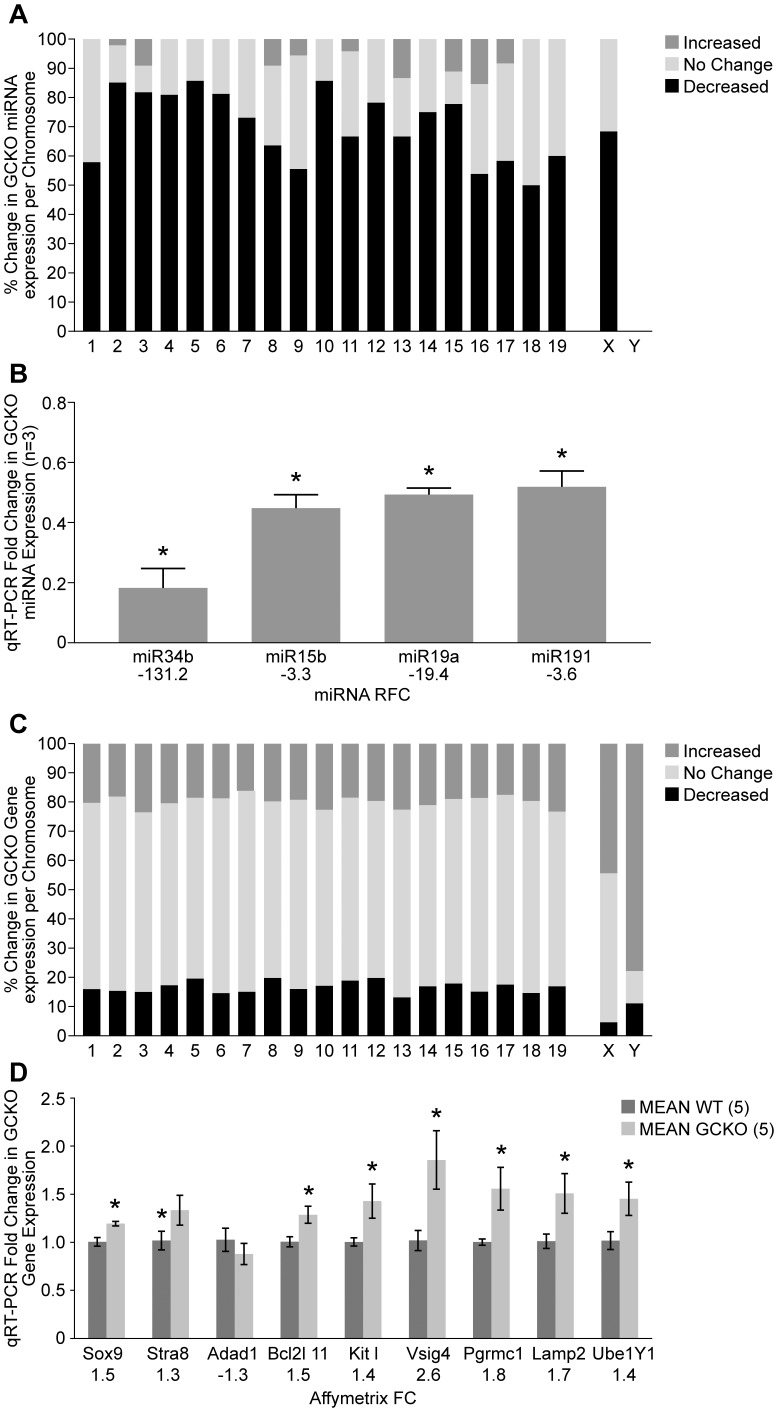
Preferential down-regulation of miRNAs and overexpression of XY genes in GCKO testis samples by P18. A) Febit miRNA arrays show 96.1% (296/308) of miRNAs encoded on all chromosomes except the Y were predominantly down regulated in GCKO testes by P18, with a small remainder (12/308 = 3.9%) upregulated. B) Validation of Febit miRNA array fold changes by qRT-PCR and primers amplifying miR-34b-3p, -15b-5p, -19a and -191. In comparison to WT (n  = 3; RFC  = 1.0), significant reductions in GCKO miRNA expression were observed using qRT-PCR and P18 RNA from GCKO testes (n = 3). C) Affymetrix ST 1.0 gene arrays showed dysregulation of 36.7% (7700/20985) of gene expression in GCKO testes with 55.7% (4290/7700) showing increased and 44.3% (3410/7700) showing decreased expression for autosomal genes. In contrast, X- and Y-linked genes were preferentially overexpressed in P18 GCKO testis samples (44.3% and 77.8%, respectively). D) Validation of Affymetrix mRNA array fold changes by qRT-PCR and primers amplifying genes highly expressed in somatic (*Sox9*) and germ cells (*Stra8, Adad1, Bcl2l11, Kitl*), and linked to X (*Vsig4, Pgrmc1, Lamp2*) and Y (*Ube1Y1*) chromosomes.

**Table 2:** Number of miRNAs and mRNAs up- or down-regulated for each chromosome in GCKO testes on P18. Data for miRNA and mRNA are expressed on a per chromosome basis and reported for those probe sets with intensities that could be scored as increased, decreased or no change in GCKO when compared to WT testes.

Chromosome Name	miRNAs Scored	Up	Down	No Change	mRNAs Scored	Up	Down	No Change
1	19	11	8	0	1212	245	194	773
2	47	40	6	1	1770	320	273	1177
3	11	9	1	1	992	233	149	610
4	21	17	4	0	1217	248	211	758
5	14	12	2	0	1206	223	237	746
6	16	13	3	0	1181	220	173	788
7	26	19	7	0	1872	302	283	1287
8	11	7	3	1	1032	204	205	623
9	18	10	7	1	1201	230	193	778
10	7	6	1	0	972	220	167	585
11	24	16	7	1	1596	294	302	1000
12	46	36	10	0	679	133	135	411
13	15	10	3	2	781	176	103	502
14	20	15	5	0	753	158	128	467
15	9	7	1	1	794	150	142	502
16	13	7	4	2	679	126	103	450
17	12	7	4	1	1012	177	178	657
18	4	2	2	0	496	97	73	326
19	5	3	2	0	719	167	122	430
X	57	39	18	0	812	360	38	414
Y	0	0	0	0	9	7	1	1
**Totals**	395	286	98	11	20985	4290	3410	13285

### Immunofluorescence Assays

Immunodetection of DICER1 was performed on 5 µm sections of P18 testes fixed in 10% neutral buffered formalin (NBF). After removal of paraffin, rehydration and antigen retrieval using sodium citrate (0.01 M, pH 6.0), sections were blocked 1 hr in 3% normal goat serum in PBS +0.05% Tween 20 (PBS-T) and incubated overnight at 4°C with rabbit polyclonal antibody against the N-terminal helicase domain of DICER1 (Sigma Prestige Antibodies, HPA000694; 1∶100). Slides were washed 3 times for 5 min in PBS-T before adding a 1∶500 dilution of goat anti-rabbit Alexafluor 568 to sections. Secondary antibody was removed after 1 h by washing slides 3 times for 5 min in PBS-T, sections were mounted with a drop of Vectashield + DAPI (Vector Laboratories, Burlingame, CA) and coverslip. Images were captured using a Nikon Eclipse E600 equipped with a digital camera and Image Pro Plus 7.0 software.

**Figure 5:**
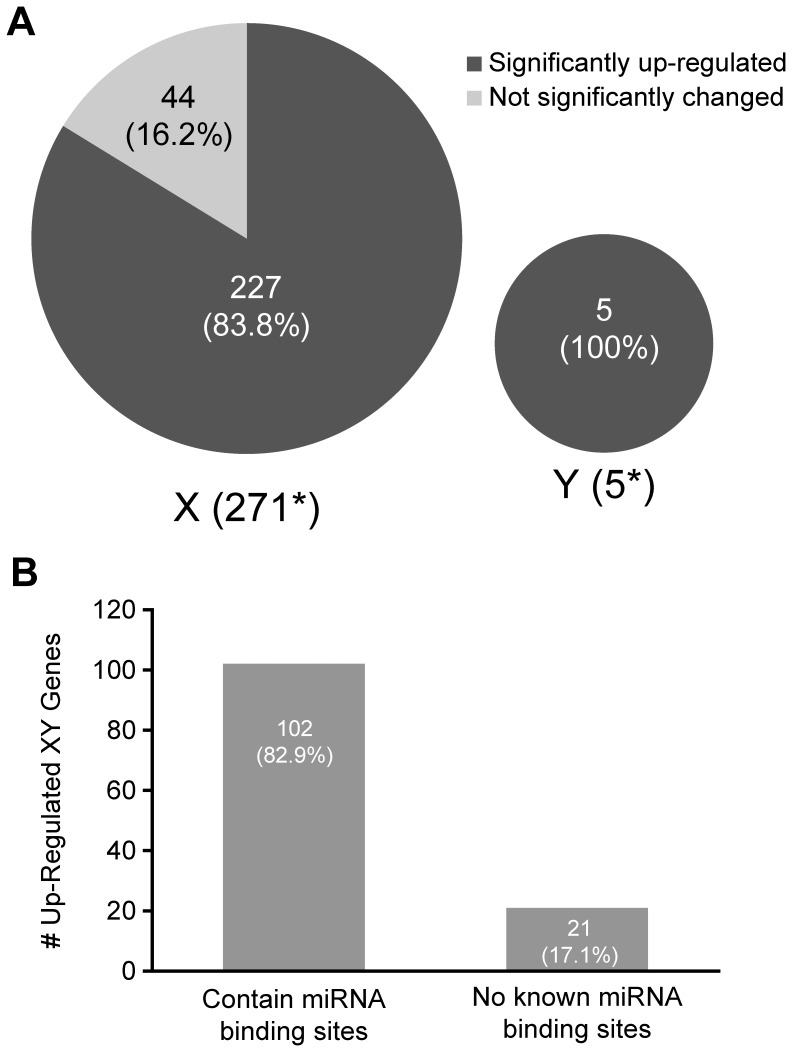
Failure of meiotic sex chromosome inactivation (MSCI) in P18 *Dicer1* GCKO testes. A) Pie charts show the number of genes that are normally silenced during MSCI that were detected by array overlaid with the percentages of X- and Y-genes in GCKO testes with expression levels that were either significantly up-regulated (83.8% and 100%, respectively) or not changed (16.2 and 0%, respectively. B) Bar graph showing the number of overexpressed (>1.5 RFC) X- and Y-genes with or without binding sites for miRNAs shown to be deregulated in P18 GCKO testes. The majority of overexpressed genes (102/123 = 82.9%) contain recognition sites for deregulated miRNAs.

### Preparation of Meiotic Cell Spreads

Meiotic cell spreads were prepared and meiotic prophase events were classified according to Cobb et al. [20]. Testes were collected from WT and GCKO mice on P22 and placed in 1 ml Dulbecco’s Modified Eagle Medium (DMEM) with L-glutamine. Following removal of the tunica, tubules were fragmented using forceps and pipetted about 10 times to remove interstitial cells. Tubules were gently washed 2 times in PBS before adding 2 ml PBS containing 0.25 mg/ml collagenase Type IV. After a short 30 second (s) incubation, tubules were rinsed 4 times with 2 ml PBS before adding 2 ml prewarmed 0.25% trypsin containing 0.125 mg/ml DNase I. Tubules were incubated 10 min at 37°C. Forty µl of soybean trypsin inhibitor (5 mg/ml) were added before pipetting tubules to a single-cell suspension. Cells were filtered through a 40 micron cell strainer (BD Falcon, Franklin Lakes, NJ) to remove debris. Cells were washed once in buffer (DMEM, 2%BSA, 10 mM EDTA) and counted. Cell concentrations were adjusted to 3×10^6^/ml and 2 µl of each cell suspension were applied to Shandon multi-well slides (Thermo-Shandon USA). Cells were spread in a circular motion over the well using the pipette tip and slides were air-dried for 5–10 min. Cells were permeabilised in cold CSK buffer (100 mM NaCl, 300 mM sucrose, 3 mM MgCl_2_, 10 mM PIPES, 0.5% Triton X-100, 1 mM EGTA and 2 mM vanadyl ribonucleoside (pH 6.8) for 10 min and then fixed 10 min in cold 4% paraformaldehyde in PBS (pH 7–7.4). Slides were rinsed once in 70% EtOH and stored in 70% at 4°C until staining with either rabbit polyclonal anti-mouse synaptonemal complex protein 3 (SYCP3) (Novus Biologicals, NB 300-231; 1∶1,000) or mouse monoclonal γH2AX (phospho S139) (Abcam, ab22551; 1∶100) and counterstained with 1∶1000 dilutions of goat anti-rabbit Alexafluor 568 (Invitrogen, A11036) or goat anti-mouse Alexafluor 488 (Invitrogen, A11001). Spermatocyte scoring criteria were: diffuse pattern of reactivity with γH2AX (phospho S139) antibody – leptotene/zygotene spermatocytes; reactivity with γH2AX (phospho S139) antibody restricted to XY body – pachytene/diplotene spermatocytes; and, no reactivity with γH2AX (phospho S139) antibody and centromeric staining with SYCP3– metaphase I spermatocytes [21].

**Figure 6:**
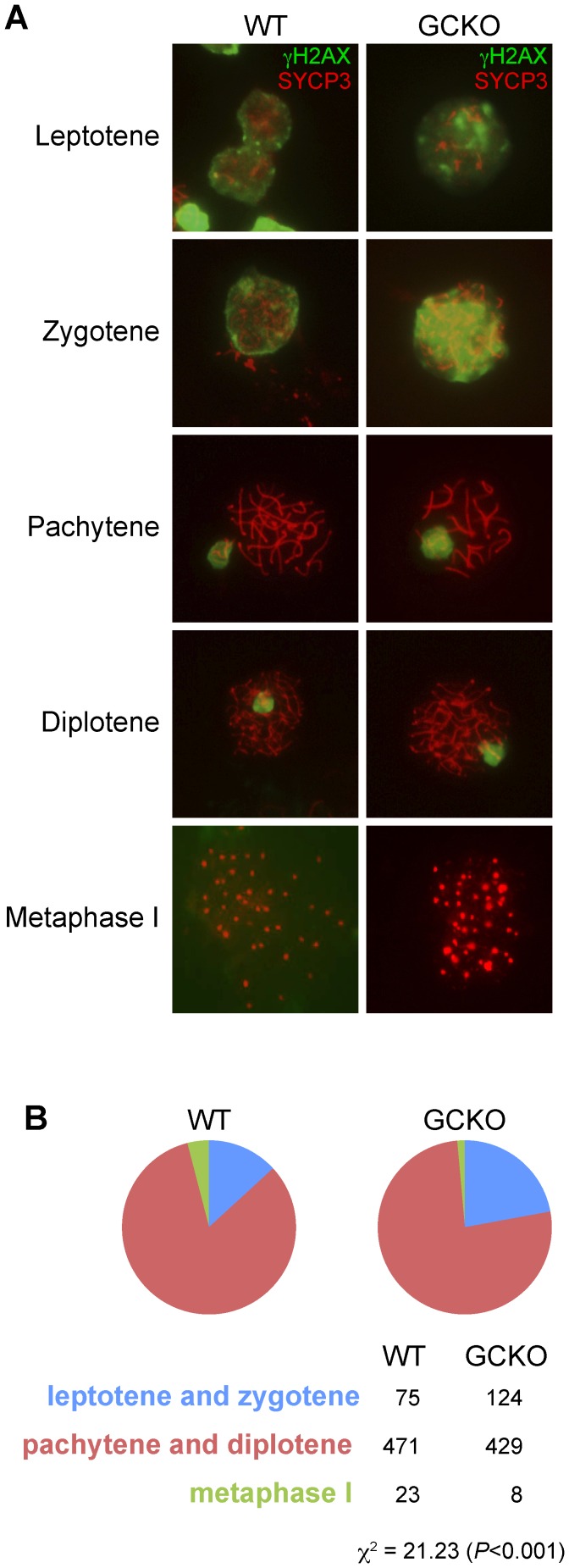
Meiotic spreads show deletion of *Dicer1* disrupts progression of meiosis I. (A) It was possible to identify the 5 sub-stages of meiosis I by combining the reactivity patterns of two antibodies against synaptonemal complex protein 3 (SYCP3) and γH2AX. SYCP3 is a protein essential for synapsis of homologous chromosomes and γH2AX localizes to double-strand breaks and XY bodies during meiosis. A total of 200 spreads were counted in testis cell preparations from WT and GCKO mice at P22. (B) Chi square analysis shows GCKO testes contained significantly higher numbers of germ cell spreads at the leptotene and zygotene stages of meiosis I and fewer spreads at pachytene, diplotene and metaphase I stages (*P*<0.001), suggesting that the loss of *Dicer1* lead to disruptions in progression through meiosis I.

### Western Blotting to Detect DICER Expression in P18 WT and GCKO Testes

Testis protein preparation and Western blotting methods were performed as described previously [22]. Following semi-dry transfer of proteins to Immobilon P membrane (Millipore Corp, Billerica, MA) the 217 kDa DICER1 protein was detected using a 1∶125 dilution of a rabbit polyclonal DICER1 antibody (Sigma Prestige Antibodies, HPA000694) and a 1∶3000 dilution of HRP-goat anti-rabbit (BioRad). Images were developed with ECL reagent (Amersham; RPN 2106).

### Gene and miRNA Expression Array Analyses at P18

Total RNA, including miRNAs, were purified from WT (n = 3) and GCKO (n = 3) testes on P18 using the mirVana™ miRNA Isolation Kit followed by analysis on the Agilent 2100 Bioanalyzer to determine quality. Following reverse transcription with random primers containing a T7 promoter sequence (Affymetrix, Santa Clara, CA), double stranded cDNA was synthesized with the GeneChip® WT cDNA Synthesis and Amplification Kit (Affymetrix). In an in vitro transcription (IVT) reaction with T7 RNA polymerase, the cDNA was linearly amplified to generate cRNA. In the second cycle of cDNA synthesis, random primers were used to generate single stranded DNA in the sense orientation. Incorporation of dUTP in the cDNA synthesis step allowed for the fragmentation of the cDNA strand utilizing uracil DNA glycosylase (UDG) and apurinic/apyrimidinic endonuclease 1 (APE 1) that specifically recognizes the dUTP and allows for breakage at these residues. Labeling occurred by terminal deoxynucleotidyl transferase (TdT) where biotin was added to the cDNA by an Affymetrix Labeling Reagent. 2.3µg of biotin-labeled and fragmented cDNA was then hybridized to the GeneChip® Mouse Gene 1.0 ST Array (Affymetrix) for 16 hours at 45°C. Post-hybridization staining and washing was performed according to manufacturer’s protocols using the Fluidics Station 450 instrument (Affymetrix). The arrays were then scanned with a GeneChip® Scanner 3000 laser confocal slide scanner.

For the miRNA arrays, the same WT and GCKO testis RNA samples were analyzed with a Geniom Realtime Analyzer (GRTA, Febit, GmbH, Heidelberg, Germany) using the Geniom Biochip miRNA *Mus musculus*. Each array contained 5 replicates of 609 miRNAs and miRNA star sequences as annotated in the Sanger miRBase 12.0. Sample labeling with biotin was carried out by microfluidic-based enzymatic on-chip labeling of miRNAs. Biotin-labeled miRNA was hybridized to the Biochip for 16 hours at 42°C. The Biochip was then washed automatically and signals were detected with the GRTA and evaluated using the Geniom Wizard Software.

### Bioinformatic Analysis of Affymetrix Gene Array and Febit miRNA Array Datasets

For the gene array data, average signal intensities for each probe set within arrays were calculated by the RMA function provided within the Affymetrix package for R using a custom (Entrez Gene) chip description file [23]. The RMA method incorporates convolution background correction, sketch-quantile normalization, and summarization based on a multi-array model fit robustly using the median polish algorithm (all samples processed together). For this experiment, one pairwise comparison was used to statistically resolve gene expression differences between WT and GCKO sample groups using the R/maanova analysis package [24]. Specifically, differentially expressed genes were detected by using *F_s_*, a modified F-statistic incorporating shrinkage estimates of variance components from within the R/maanova package [25] [24]. Statistical significance levels of the pairwise comparison were calculated by permutation analysis (1000 permutations) and adjusted for multiple testing using the false discovery rate (FDR), q-value, method [26]. Differentially expressed genes were declared at an FDR q-value threshold of 0.05.

The miRNA array data were derived from Febit microarrays after background subtraction (subtracting median negative control intensities from each array). The background-subtracted data were transformed by adding a constant of one to all intensities and applying a log2 transformation. Any probe which had a median value of zero (zero intensity in at least two arrays in each sample group) was removed from the dataset. These probes were deemed to have no expression in either sample group. Upon filtering the probe sets, the density and distribution of intensities were examined. Upon review, sample M36 (GCKO sample) was found to have a distribution of intensities similar to WT samples. Data from sample M36 were removed based upon these criteria. All intensity data were reexamined for the remaining five arrays. Probes which had a median of zero (zero intensity in at least two arrays) for the WT samples and a minimum intensity of zero in at least one array for the mutant samples were removed. These probes were deemed to have no expression in either sample group. Upon filtering such probe sets, the density and distribution of intensities within sample groups were relatively equal. Quantile normalization was performed within sample groups given the distinct difference in intensity distributions between sample types. The normalized data were used for microarray analysis in R/maanova. One pairwise comparison was used to statistically resolve miRNA expression differences between WT and GCKO groups using the R/maanova analysis package [24]. Differentially expressed miRNAs were detected using *F_s_,* a modified F-statistic, incorporating shrinkage estimates of variance components from within the R/maanova package. Statistical significance levels of the pair-wise comparisons were calculated by permutation analysis and adjusted for multiple testing using the false discovery rate (FDR) q-value method [26]. Differentially expressed miRNAs were declared at an FDR q-value threshold of 0.05.

### Analysis of Putative MSCI Targets in P18 GCKO Testes

Probe IDs representing a total of 369 MSCI genes identified in Namekawa, et al. [27] were downloaded and annotation updated at www.affymetrix.com. Un-annotated, non-specific, and replicate probes were removed which resulted in a list of 304 (299 X-linked and 5 Y-linked) individual genes representing putative MSCI targets. This gene list was then cross-referenced with all X and Y genes detected in the array analysis of P18 WT and GCKO testes resulting in a list of 276 (271 X-linked and 5 Y-linked) genes. This gene list was then queried against the arrays to determine the expression pattern in GCKO relative to WT testes. Significance was set at q <0.05.

### Identification of miRNA Recognition Sites in Sex-chromosome Linked Genes Up-regulated in P18 GCKO Testes

The 3′ UTRs of genes greater than 1.5-fold up-regulated relative to WT in P18 GCKO whole testes were queried for *Mus musculus* miRNA recognition sites using the Microcosm Target database (http://www.ebi.ac.uk/enright-srv/microcosm/htdocs/targets/v5/). Genes encoding multiple isoforms were excluded to eliminate the possibility of multiple isoforms being present in the testis. Identified miRNA recognition sites were then classified into different groups: absent in the miRNA arrays of P18 WT and GCKO, not significantly changed, significantly down-regulated, or significantly up-regulated. Overlapping miRNA recognition sites of miRNAs detected in the arrays were eliminated using a moving window method, selecting for sites with the highest score and ensuring maximal site coverage. Up-regulated genes were then classified as either containing or not containing miRNA recognition sites of miRNAs down-regulated in the GCKO testis relative to WT.

### Quantitative Real-Time PCR to Validate miRNA and Gene Expression Profiles

Relative fold changes for GCKO miRNA and mRNA arrays were verified using qRT-PCR. Total RNA was isolated from WT and GCKO testes collected on P18 using TRIZOL Reagent (Invitrogen) following manufacturer’s instructions. RNA was quantified using a NanoDrop ND1000 spectrophotometer and cDNA was prepared from 1 µg total RNA using the SuperScript III synthesis kit (Invitrogen). Quantitative PCR validation of miRNA expression in P18 GCKO testes was performed using the TaqMan MicroRNA Assay system (Applied Biosystems) following manufacturer’s suggestions. Briefly, 10 ng of total RNA were reverse transcribed using primers specific to miRs highly expressed in male germ cells (19a-3p and 34b-3p) [11], [28], [29], testis (15b-5p and 191-5p) [4] and U6 snRNA (assay IDs 2618, 2299, 390, 395, and 1973). The resulting products were amplified and quantified using TaqMan Universal PCR Master Mix II, No UNG (Applied Biosystems) and target specific primers on an ABI 7500 Real Time PCR System. Quantitative RT-PCR to validate gene expression data was performed using SYBR Green PCR master mix (Applied Biosystems, 4309155) and primers for somatic (*Sox9*) and germ cells (*Stra8, Adad1, Bcl2l11, Kitl*) and over-expressed X- (*Vsig4, Pgrmc1, Lamp2*) and Y- *(Ube1Y1)* genes. Primer sequences are listed in Table 1. qPCR reactions were performed on an ABI 7500 Real Time PCR System and the expression of each gene was normalized to beta actin (*Actb*). MiRNA and mRNA expression changes were determined using the ddCt method as described for litter-matched WT and GCKO pairs [30]. Significant differences (*P*≤0.05) in expression levels were evaluated by *t*-test using JMP-10 software.

## Results

### Germ Cell Knock Out (GCKO) Male Mice are Infertile

In comparison to littermate controls, GCKO mice grew normally to adulthood as evidenced by similar body weights at P22, 5 and 8 weeks of age (Figure 1A). However, significant reductions in testis weight were evident in GCKO males at the 5 and 8 week time points (P<0.0001 and P  = 0.004, respectively) (Figure 1A). Compared with WT controls, sperm numbers showed ∼94% reduction in the epididymal ducts of GCKO males by 8 weeks (Figure 1B). Phase contrast microscopy showed >90% of sperm present in 8 week GCKO males have malformations of either the head (round-, double-, small-heads) or tail (kinked, thickened). Furthermore, despite an ability to mate and produce copulation plugs, GCKO males were unable to sire litters when bred with WT females (Figure 1C). Hematoxylin-eosin (HE) staining of WT and GCKO testis seminiferous tubules at 5, 8 and 10 weeks showed lumen enlargement, pyknosis and progressive loss of elongating and elongated spermatids in GCKO tubules (Figure 2A). Mature spermatozoa were rare in the epididymides of GCKO males by 8 weeks and remained scant at 10 week (Figure 2B).

### *Dicer1* Deletion Reduces *Dicer1* Transcript and Protein Expression by P18

The germ cell-specific *Stra8-*icre transgene is only expressed in males beginning at P3 in type A spermatogonia through preleptotene spermatocytes [18]. P18 was selected as the time point for the following analyses because the testis cellularity was histologically similar between the WT and the GCKO males. Using qRT-PCR, a six-fold reduction in *Dicer1* RNase III transcripts was measured in RNA samples from GCKO testes (*P*  = 0.018) providing evidence of cre-mediated deletion of the *Dicer1* conditional allele (Figure 3A). Western blotting demonstrated significantly reduced levels of DICER1 in the GCKO testis protein lysates (Figure 3B). The same antibody was used to localize DICER1 in P18 WT and GCKO testis sections (Figure 3C). In WT sections, DICER1 was present in the cytoplasm of Sertoli cells, spermatogonia and pachytene spermatocytes. In the GCKO sections, DICER1 was observed only in Sertoli cells and spermatogonial stem cells near the tubule basement membrane (Figure 3C). *Dicer1* deletion was not expected in Sertoli cells or spermatogonial stem cells as *Stra8*-icre would not be active in these cell types. No differences in cellular composition between P18 WT and GCKO testes were observed by histology (Figure 3D). Therefore, selective loss of *Dicer1* in spermatogonia of GCKO testes reduced the expression of DICER1 before altering cellular composition of the tubule.

### miRNA and Transcriptome Deregulation were Evident at P18

As no differences in cellular composition between P18 WT and GCKO testes were observed histologically (Figure 3D), Febit miRNA arrays were used to compare miRNA expression profiles at P18 in WT (n = 3) and GCKO testes (n = 2). In comparison to WT testes, 75.2% (297/395) of miRNAs showed significant deregulation. Among these, 96.3% (286/297) of miRNA genes on all chromosomes except the Y were significantly down-regulated, while 3.7% (11/297) miRNA genes were overexpressed in GCKO testes (Figure 4A). To date, miRNA genes have not been identified on the Y chromosome (Table 2) [1]. To confirm miRNA array findings, expression levels of four mature miRNAs (miR-34b-3p, 15b-5p, 19a and 191) were measured using qRT-PCR. Based on the array results, the relative fold changes (RFCs) of these miRNAs in the GCKO testes compared to WT were −131.2, −3.3, −19.5 and −3.6, respectively. The qPCR results confirmed the down-regulation of all four miRNAs (Figure 4B, all P≤0.05).

Protein-coding transcripts of WT (n = 3) and GCKO (n = 3) testes at P18 were compared by mouse Affymetrix 1.0 ST gene arrays. Of the probe sets defined as present, 36.7% (7700/20,985) showed differential expression in the GCKO testes. Approximately half of all differentially expressed transcripts (4290/7700 = 55.7%) were up-regulated and the remainder (3410/7700 = 44.2%) were down-regulated (Figure 4C). In comparison to autosomal genes, disproportionately high percentages of X- and Y-linked genes were overexpressed in the GCKO testis (19.8% of autosomal genes vs 44.3% of X-linked and 77.8% of Y-linked genes). Overexpression of X-linked (*Visg4*, *Pgrmc1*, and *Lamp2*) and Y-linked (*Ube1y1*) genes was validated by qRT-PCR using RNA isolated from WT (n = 5) and GCKO (n = 5) testes using samples other than those used for array analyses. The genes specifically expressed in Sertoli cells (*Sox9*), early undifferentiated spermatogonia (*Stra8*), round spermatids (*Adad1*), and germ cells (*Bcl2l11, Kitl*) were also verified by qRT-PCR (Figure 4D).

Due to the preponderance of mis-expressed X and Y-linked genes in the GCKO testes we examined sex chromosome inactivation in GCKO testes by comparing a list of previously published genes thought to undergo MSCI [27] with X and Y-linked genes expressed in GCKO testes. Figure 5A shows the number of up-regulated X- and Y-linked genes in the GCKO testis that overlapped with the gene list (271 and 5, respectively). A total of 227/271 (83.8%) and 5/5 (100%) of the genes on the X and Y chromosomes, respectively, were over-expressed in the GCKO testes relative to WT at P18. To characterize possible effects of miRNAs on these transcripts, the number of up-regulated X- and Y-linked genes with and without potential miRNA recognition sites was determined. It was assumed that transcripts up-regulated, yet having no miRNA binding sites in the 3′UTR, may be subject to indirect effects of the miRNAs, whereas those with binding sites for miRNAs may be subject to translation inhibition and/or transcript turnover. Figure 5B shows a subset of overexpressed (>1.5 RFC) X and Y-transcripts with or without binding sites for deregulated miRNAs. The majority of up-regulated genes (102/123 = 82.9%) contain recognition sites for deregulated miRNAs while 17.1% (21/123) of up-regulated genes contain no known binding sites for deregulated miRNAs. These findings suggest that the disruption of miRNA biogenesis in the GCKO testis may contribute to overexpression of genes encoded on the sex chromosomes that should be silenced during MSCI.

### Deletion of *Dicer1* Delays Meiotic Progression in GCKO Germ Cells

To determine if spermatogenic failure in GCKO testes might be explained by delays in meiotic progression, we identified germ cells in sub-stages of meiotic prophase in P22 WT and GCKO testes. Chromosomes were visualized in meiotic spreads with a synaptonemal marker, SYCP3, and sex body marker, phosphorylated histone H2AFX (γH2AX). The antibody against SYCP3 recognizes a component of the axial element of the synaptonemal complex, while the antibody γ-H2AX marks double strand breaks in leptotene spermatocytes and also detects the XY bodies of pachytene spermatocytes [31], [32], [33], [34]. As shown in Figure 6A, double-labeling of spermatocytes with SYCP3 and γ-H2AX antibodies made it possible to score the proportion of leptotene, zygotene, pachytene, diplotene and metaphase I stages in WT and GCKO meiotic spreads. Figure 6B shows that in comparison to WT, significantly more cells were identified at the leptotene and zygotene stages and fewer were identified at pachytene, diplotene or metaphase I stages in GCKO speads (*X^2^* = 21.2; *P*<0.001). The higher number of early meiotic cells (leptotene and zygotene) and the reduced number of late meiotic cells (pachytene, diplotene and metaphase I) in the GCKO mutant suggest the loss of *Dicer1* disrupted spermatocyte progression through meiosis I.

## Discussion

Here, we investigated the deletion of *Dicer1* in mouse postnatal male germ cells to determine how disruptions in miRNA processing may contribute to male infertility. We report that deletion of *Dicer1* from postnatal spermatogonia resulted in spermatogenic failure following large-scale loss of miRNA processing, deregulation of the testis transcriptome and preferential overexpression of sex chromosome genes. Chromosomal spreads prepared from GCKO germ cells and overexpression of genes that undergo MSCI provided evidence that deletion of *Dicer1* may impair meiotic progression as well as post-meiotic spermiogenesis.

Earlier reports have characterized spermatogenic failure following *Dicer1* removal from postnatal male germ cells. Selective ablation in E18-P3 spermatogonia to post-meiotic spermatids was accomplished by crossing mice carrying floxed *Dicer1* alleles with transgenic mice expressing cre recombinase under the control of *Neurog3*, *Ddx4*, *Stra8* or *Pgk2* promoters [15], [16], [35], [36]. Severity of the reproductive phenotypes depended on the stage at which *Dicer1* was inactivated, with the most marked effects following *Dicer1* deletion from E18-P3 spermatogonia which resulted in reduced testis weight and sperm count and sterility. Histology of adult tubules revealed germ cell apoptosis, organizational defects and an absence of elongated spermatids. Meiotic progression defects were observed in *Ddx4*-cre;*Dicer1* ^−/−^ testes as were head and tail abnormalities. *Dicer1* excision in male germ cells led to reduced abundance of a subset of miRNAs (miR-34c and miR-184) and significantly increased transcript levels of SINE B1 and B2 transposable elements [16]. We extended these findings to include comparisons of 410 testis-expressed miRNAs and 20,985 mRNAs.

During meiosis in male mammals, the X and Y chromosomes behave differently from autosomes. Autosomal homologues become tethered, or synapsed along their entire length, whereas the nonhomologous X and Y chromosomes remain largely unsynapsed. This asynapsis is essential for driving meiotic sex chromosome inactivation (MSCI) [37]. Recent evidence suggests that MSCI is essential for male fertility [38]. To determine if *Dicer1* deletion impairs MSCI, we conducted a bioinformatics study and found that 84% of X and 100% of Y genes overexpressed in GCKO testes overlap with sex chromosome genes reported to undergo MSCI [27]. Interestingly, among the genes overexpressed in GCKO testes, we observed up-regulation of *Zfy2*, a gene that when not silenced on the Y during meiosis is capable of inducing pachytene arrest [38]. Moreover, 83% of X- and Y-linked genes overexpressed in the GCKO testis contained binding sites for deregulated miRNAs suggesting that these miRNAs may actively participate in regulating levels of transcripts encoded on sex chromosomes and silenced during MSCI. Alternative interpretations of this data are that MSCI is intact in the GCKO testis and overexpression of X-and Y-linked genes may be explained by either by an increase in transcript stability or by enrichment of pre-MSCI spermatocytes. A paper published during the preparation of our report provides support for both of these possibilities [39].

Endogenous-siRNAs (endo-siRNAs) are abundantly expressed in the testis and, similar to miRNAs, have cytoplasmic roles as posttranscriptional regulators and function in male germ cell development [36]. Knock-out studies in mice have shown that the microprocessor complex (DROSHA-DGCR8) is essential for processing precursor miRNAs from primary miRNA transcripts in the nucleus but is not required for endo-siRNA biogenesis. DICER1 activity however, is required for processing both endo-siRNAs and miRNAs. Therefore, by deleting *Dicer1* from male germ cells, endo-siRNA levels may also be reduced and thus confound the etiology of the *Stra8*-icre;*Dicer* infertility phenotype. Arguing against this possibility is the finding that both *Stra8*-icre;*Dicer* ^lox/lox^ and *Stra8*-icre;*Drosha* ^lox/lox^ male mice are infertile with oligozoospermia or azoospermia due to constant depletion of pachytene spermatocytes and spermatids. If endo-siRNAs were principally responsible for the Dicer1 infertility phenotype, then *Drosha* KO males should have been fertile because levels of endo-siRNAs were unchanged. The fact that fertility was not rescued in the *Drosha* KO mice suggests that broad-based reduction in the levels of miRNAs in our model may better explain the *Dicer1* knockout infertility phenotype. Interestingly, female *Dicer* and *Dgcr8* knockout models suggest that miRNA and endo-siRNAs have reversed importance in regulating mouse oocyte and early embryo gene expression [40].

Infertility affects about 15% of couples worldwide [41]. Studies in model organisms have revealed several of the molecular and genetic pathways that regulate fertility [42], [43]. In mice, more than 200 genes participate directly or indirectly in fertility and provide possible targets for treatments and contraception [41]. In humans, approximately 25% of infertility in men is attributed to deletions of the Y chromosome or aneuploidy of the X chromosome [44], [45]. The remainder of human infertility is unexplained and thought to be genetic; however, studies to identify point mutations responsible for spermatogenic failure have left many unresolved questions. Progress may be challenged by the fact that, as with mice, hundreds of human genes regulate fertility and the contribution of a small number of mutations may be insignificant [46], [47]. The importance of our study to the origins of male infertility is the finding of global disruption in regulatory homeostasis of meiotic and post meiotic genes following large-scale down-regulation of miRNAs. Spermatogenic failure in our model may be better explained by aberrant regulation of gene expression, perhaps contributing to transcript instability and disruption of MSCI rather than individual gene mutations.
